# Determination of Interesting Toxicological Elements in PM_2.5_ by Neutron and Photon Activation Analysis

**DOI:** 10.1155/2013/458793

**Published:** 2013-06-25

**Authors:** Pasquale Avino, Geraldo Capannesi, Francesco Lopez, Alberto Rosada

**Affiliations:** ^1^Air Chemical Laboratory, DIPIA, INAIL Settore Ricerca, Via IV Novembre 144, 00187 Rome, Italy; ^2^UTFIST-CATNUC, ENEA, Via Anguillarese 301, 00060 Rome, Italy; ^3^Dipartimento di Agricoltura, Ambiente Alimenti (DIAAA), University of Molise, Via De Sanctis, 86100 Campobasso, Italy

## Abstract

Human activities introduce compounds increasing levels of many dangerous species for environment and population. In this way, trace elements in airborne particulate have a preeminent position due to toxic element presence affecting the biological systems. The main problem is the analytical determination of such species at ultratrace levels: a very specific methodology is necessary with regard to the accuracy and precision and contamination problems. Instrumental Neutron Activation Analysis and Instrumental Photon Activation Analysis assure these requirements. A retrospective element analysis in airborne particulate collected in the last 4 decades has been carried out for studying their trend. The samples were collected in urban location in order to determine only effects due to global aerosol circulation; semiannual samples have been used to characterize the summer/winter behavior of natural and artificial origin. The levels of natural origin element are higher than those in other countries owing to geological and meteorological factors peculiar to Central Italy. The levels of artificial elements are sometimes less than those in other countries, suggesting a less polluted general situation for Central Italy. However, for a few elements (e.g., Pb) the levels measured are only slight lower than those proposed as air ambient standard.

## 1. Introduction

The distribution of elements in airborne particulate matter is fundamentally determined by resuspension of various substances of natural and/or artificial origin, by their type of circulation due to the meteorological events, and by the chemical behavior of the elements.

Anthropogenic activities introduce into the environment materials that give rise to increasing levels of many substances which may endanger the environmental quality and represent a hazard to human health.

Major attention has been given to those elements who are more liable to alter the environment and endanger human health.

Trace elements in airborne particulate are an important issue for the implications regarding the health effects of some elements (e.g., Cd, Hg, and Pb). Furthermore, the airborne particulate matter pollutant, especially the distribution and multielemental composition of fine particles with diameter <2.5 *μ*m (PM_2.5_), is considered one of the most difficult tasks in environmental chemistry for its complex composition and implications that complicate notably the behavior comprehension [1–5].

Since pollution from trace elements has been considered, the evaluation of background levels due to natural pathways of circulation seems the proper preliminary action to be undertaken. This study was therefore extended as far back as possible in time (from 1965 until 2000) in order to analyze the trend of airborne concentration of pollutant elements in connection with the industrial and lifestyle growth during the entire period.

Instrumental nuclear techniques are widely used in this field [4, 6–28] as they represent the most reliable method for analyzing trace and/or ultratrace elements in air particulate PM_2.5_. Instrumental Neutron Activation Analysis (INAA) as well as Instrumental Photon Activation Analysis (IPAA) have been employed in this work to measure interesting toxicological elements. In particular, over metals such as As, Cd, Cr, Hg, Pb, Sb, and Zn considered of greater health concern, other elements of relevant environmental interest or less considered previously were measured.

## 2. Experimental Part

### 2.1. Sampling

For particulate matter sampling a dichotomous sampler (mod. SA 241, Graseby-Andersen) operating at 16.7 L min^−1^ was used. This sampler has a PM10 size selective inlet and separates the aerosol into fine (aerodynamic diameter, *D*_*a*_, <2.5 *μ*m) and coarse (2.5 *μ*m < *D*_*a*_ < 10 *μ*m) fractions. Particulate matter was collected on polymethylpentene ringed, 2.0 *μ*m pore size, 37 *μ*m, Teflon membranes (Gelman, type R2PJ). This sampler has been designed as reference by USA-EPA. The PM_2.5_ samples were stored in box at controlled conditions (atmosphere and temperature).

About 300 air samples have been collected in downtown Rome (41°53′54′′N, 12°29′43′′E), in an area characterized by high presence of anthropogenic activities. The sampling was 24-hour long for each filter. All the storage and handling sample treatment were carried out at the ENEA and INAIL laboratories.

### 2.2. INAA and IPAA Analyses

Among the different analytical methodologies available for element determination we used nuclear approach for its important analytical properties. In fact, the various analytical techniques (spectroscopy, electrochemistry, and chromatography) do not permit to have the maximum information because of their limitations. INAA is well known as reference analytical technique because all the experimental steps are totally traceable and there is absence of physical-chemical sample manipulation reducing the positive and/or negative artifacts formation. Furthermore, because of its high sensitivity, multielemental character allowing the determination of about 40 elements with a good Limit of Detection (LOD) (Table 1) and accuracy, the INAA has surpassed other instrumental methods for trace/ultratrace metal and rare earth element analysis: a comparative study [29], however, has pointed out that INAA is blank free and especially suitable for the analysis of reference materials [30]. Further, we used IPAA as a complementary technique for determining elements: in particular, Pb, Tl, and Zr which are difficult to determine by INAA, are important from toxicological and environmental point of view.

Table 1 shows the nuclear data (as product nuclide, half-life and energy peak emission) and LOD (expressed as ng m^−3^) of each element investigated in this study by means of INAA and IPAA. It should be noted the very low LODs reached by nuclear techniques in relation with other analytical methodologies [29, 31].

INAA: samples, blank, and standards, put in nuclear-grade polyethyene cylinders (Kartell), were irradiated at a neutron flux of 2.6 × 10^12^ n × cm^−2^ × s^−1^ for 32.55 h in rotatory rack “Lazy Susan” of the nuclear reactor Triga Mark II of the ENEA-Casaccia Laboratories [32].

For the analysis primary and secondary standards were used. Primary standards (Carlo Erba, Milano, Italy) were As, Cd, Co, Cr, Cs, Fe, Hg, La, Ni, Sb, Se, Sm, and Zn whereas as secondary standards United States Geochemical Survey (USGS) nn. 1, 4, 6 and the Coal Fly Ash (NIST) n. 1633a were used.

**Figure 1:**
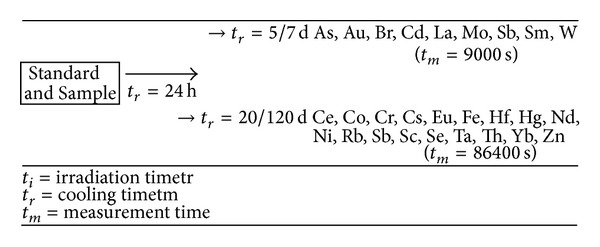
Scheme for INAA analysis of standards and samples.

After irradiation, *γ*-ray spectrometry measurements of different durations (Figure 1) were carried out using a HPGe detector (FWHM 1.68 keV at 1332 keV) connected to a multichannel analyzer equipped with software packages for a *γ*-spectra analysis.

A first measurement series was performed 5/7 days after the end of irradiation with measurement times of 3000 and 9000 s for sample for determining As, Au, Br, Cd, La, Mo, Sb, Sm, and W [31]. The second series was performed 20/120 days after the end of irradiation with measurement times of 24–100 h for sample for determining Ce, Co, Cr, Cs, Eu, Fe, Hf, Hg, Nd, Ni, Rb, Sb, Sc, Se, Ta, Th, Yb, and Zn [31].

IPAA: samples, blank, and standards (NIST SRM 1571) were irradiated in the photon beam of the INFN Frascati National Laboratory Linear Accelerator (LINAC) at an average beam current of 40 *μ*A, maximum electron energy of 300 MeV, and a W converter of 0.3 mm thickness.

Two series of measurements were carried out: after 36/70 hours Zr and Pb were measured for 2 hours whereas after 20 days from irradiation As and Tl were counted for 4 hours.

## 3. Results and Discussion

### 3.1. INAA Quality Assurance and Quality Control (QA/QC)

An important task was devoted to the investigation of Quality Control (QC) and Quality Assurance (QA) of the methodology used in this study. For these goals we performed irradiations of primary and secondary reference standard materials (RSMs) for matching the entire analytical methodology and for minimizing and/or avoiding matrix effects, respectively. About the comparison between our data and certified values it should be noted that the very low levels of some elements (<1 *μ*g g^−1^) can affect the measurements in terms of precision and/or accuracy: for an element having mean value <500 *μ*g g^−1^, result can be considered as “good” if coefficient of variation (CV%) <20%, “acceptable” if CV% between 20% and 30%, and “unsatisfactory” if CV% >30%.  Table 2 shows this analytical quality control of the INAA data. In particular, this control was performed through an intercomparison campaign for 14 elements promoted by IAEA on air filter samples among different worldwide laboratories (130) using both spectrochemical (colorimetry, fluorescence, X-ray fluorescence, infrared spectrometry, atomic absorption and emission spectrometry, ICP-AES, and ICP-MS), electrochemical (polarography, voltammetry), and nuclear (INAA, isotopic dilution, beta counting) analytical techniques. For each element in the table are reported our values determined by INAA (“measured value”) and the “certified value”: the last column (“average value”) reports the value averaged among all the determinations performed by different laboratories interested in the measurements. As it can be noted, the agreement between INAA and the real value is quite good except for some elements such as Ba, U, and Zn. For Ba and U this discrepancies can be due to the difficulty to analyze such kind of elements even if for Ba our “measured value” and the “average value” are quite similar. For Zn the situation is little bit different: in fact, the “certified value” (152 *μ*g g^−1^) falls into the INAA value range (132 ± 18 *μ*g g^−1^).

### 3.2. Particulate Matter Results

In Table 3 for each element both the summer and winter average levels are reported as well as the maximum and minimum values for each season measured in PM_10_ samples by INAA and IPAA. It may be noted that elements of artificial origin show winter concentration levels higher than the summer ones, probably owing to an enhanced production in the winter period; in contrast elements of natural origin show summer concentration levels higher than the winter ones, possibly as a consequence of an increased resuspension of soil matter in summer.

Such results are obviously strictly related to the general meteorological characteristics of Italy and therefore contain some peculiarity. If they are compared to similar results obtained in other countries under different meteorological conditions, it can be seen that they agree fairly well for the pollutant elements, whereas for most elements of natural origin there are sensible differences that may be related to the geomorphological and meteorological characteristics. In fact, the higher values found in this study for Al, Cs, Na, Rb, Th, Ti, U, and rare earths are to be related to the element content of the volcanic rocks which are very widespread in Latium [33, 34].

As a very simple approach to the element behavior, they were grouped into three categories according to the value of the ratio (*R*) of summer to winter average levels (Table 4). The first group includes elements whose *R* is greater than 2; the second group elements whose *R* is less than 2 but greater than 0.5; the last group includes elements whose *R* is less than 0.5.

Elements of natural origin are found in the first group only, while elements of both natural and anthropogenic origin are present in the second group. The third group includes only pollutant elements (Cd, Cr, Mo, Ni, Pb, V, and Zn).

After, we collected PM_2.5_ aerosol samples in downtown Rome.  Table 5 shows average concentration values, minimum and maximum levels and standard deviation of the elements determined in the PM_2.5_ fraction, whereas the correlation coefficients of the analyzed elements are reported in Table 6. It should be noted that many elements cannot be detected in these samples: the main reasons depend on both the granulometric size fraction, 2.5 *μ*m, and the very low levels reached by some elements (e.g., Nd is below LOD). Basically, the concentration levels of the elements are low: the situation is good regarding the exposition to potential toxic elements. Furthermore, a wide scattering between the correlation coefficients can be noticed: only As, Co, Fe, Sc, Sb, and Se are highly correlated (0.7 < correlation coefficient < 1, marked in bold in Table 6) whereas Au, Ba, Br, Ce, Cr, Cs, La, and Sm result scarcely correlated. This behavior can be expected considering the chemical-physical properties of the elements and the granulometric size (<2.5 *μ*m).

### 3.3. The Enrichment Factor (EF) Application

**Figure 2:**
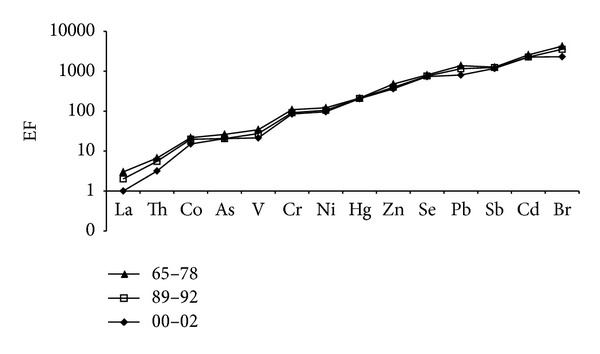
EF trend comparison of selected trace elements in PM10 fraction calculated using La as normalizing element.

In order to investigate a retrospective study of elements in PM_10_ and their evolution in relationship with the natural or anthropogenic origins, Table 7 reports the levels of selected elements collected in the last 4 decades: the data obtained show a decrease ranging between 24% (Co) and 91% (La), except for Hg, Sb, and Se. This may be attributed to the technological growth during the entire period and to the adoption of anti-pollution system in domestic heating and in industrial plants. For a better knowledge of this evolution and, especially, of the element origin, we have calculated the enrichment factors (EFs) with respect to the element abundance in the upper continental crust. Elements with EF values much higher than 1 can be considered of noncrustal origin and may be attributed to long-transport phenomena from other natural and/or anthropogenic sources. The EFs have been calculated in agreement to the equation reported in Bergamaschi et al. (2002) [14] and La as normalizing element.  Figure 2 shows the EF trend for selected elements in PM_10_ during four decades. As can be noted, all the elements may be attributed to long-range transport phenomena from other natural and/or anthropogenic sources: this behavior is common to all the period studied even if a very light decreasing trend can be evidenced from 1970 to 2002. Looking at Figure 2, three element groups can be identified according to their EFs: La and Th ranging between 1 and 5; Co, As, V, Cr, and Ni ranging between 1 and 100; Hg, Zn, Se, Pb, Sb, Cd, and Br ranging between 200 and 2500. Finally, some specific considerations can be extrapolated: the high EF values found for Br (and Pb as well) by both elaborations could be attributed to the use of leaded gasoline (cars with leaded gasoline are still present at the end of “nineties”); the sources of As, Pb, Sb, and Zn would be looked for among the various anthropogenic activities in the Rome area and particularly Sb and Zn could be attributed to traffic origin being essential components of antifriction alloys and car tires.

**Figure 3:**
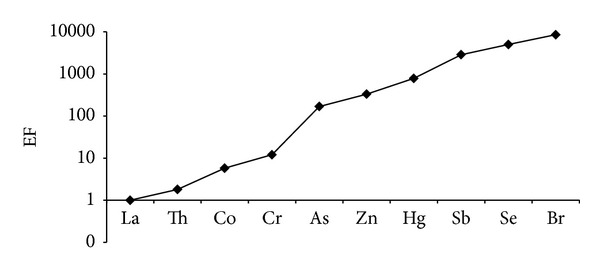
EFs of selected elements in PM_2.5_ fraction calculated using La as normalizing element.

Finally, a same approach has been performed to elements investigated in the PM_2.5_ fraction, even if no historical data series are available.  Figure 3 reports the results obtained on the PM_2.5_ fraction: Co, Cr, As, Zn, Hg, Sb, Se, and Br show EF values ranging between 5 and 8500, respectively. It should be noted that the EFs in PM_2.5_ fraction are more elevated than in PM_10_ fraction, especially for Br, Se, Sb, and Hg: this could be due to the different granulometric size and the different penetrating abilities of such elements. This occurrence can be an index of the different bioavailability of an element series present in PM_2.5_ fraction compared to PM_10_ fraction. As reported above, the higher EF value, found for Br and attributed to the use of leaded, is more evident in this fraction.

## 4. Conclusion

The experimentation has been addressed for getting the maximum analytical informative ability from the single sample determinations. The INAA and IPAA techniques have allowed to reach such elevated sensibility/accuracy levels to furnish discreet values for elements present at very low concentrations (trace and ultratrace levels). Particular attention has been devoted to reach elevated degrees of precision and accuracy for each determined element so that to increase its significance. In fact, for some elements such as Co, Se, and Th, this is the first determination in literature for Rome.

For some elements of geochemical origin (i.e., La, Th) the results reported can be considered representative of the urban area of Rome. The element concentrations determined in this study do not show a significant level of attention from a toxicological point of view taking into account that the simple measurements of the total airborne concentration of a metal may not be representative of its potential to participate in processes deleterious to health. Only few elements show a very good distribution between the two fractions whereas the greater part shows a distribution more elevated in the PM10 fraction than in the PM_2.5_ fraction whereas EFs show an opposite trend. Finally, Co, Hg, and Zn (basically, three anthropogenic elements) show no predominant distribution between the two granulometric mass.

## Figures and Tables

**Table 1:** Nuclear data and LOD of elements by INAA and IPAA (m: minutes; h: hours; d: days; y: years). LODs calculated according to [30].

Element	Product nuclide	Half life	*γ*-Ray used (keV)	LOD (ng m^−3^)
*INAA *				
Ag	^ 110m^Ag	253.0 d	657.8	0.06
Al	^ 28^Al	2.3 m	1778.9	200
As	^ 76^As	26.3 h	559.2	1
Au	^ 198^Au	2.70 d	411.8	1
Ba	^ 131^Ba	11.5 d	496.3	5
Br	^ 82^Br	1.47 d	776.5	0.5
Ca	^ 49^Ca	8.8 m	3083.0	200
Cd	^ 115m^In	53.0 h	336.6	0.3
Ce	^ 141^Ce	32.38 d	145.4	0.05
Cl	^ 38^Cl	37.3 m	1642.0	100
Co	^ 60^Co	5.272 y	1332.5	0.01
Cr	^ 51^Cr	27.7 d	320.0	0.2
Cs	^ 134^Cs	2.062 y	795.7	0.02
Eu	^ 152^Eu	12.7 y	1408.0	0.01
Fe	^ 59^Fe	45.1 d	1099.2	50
Hf	^ 181^Hf	42.4 d	482.2	0.01
Hg	^ 203^Hg	46.9 d	279.0	0.02
I	^ 128^I	25.0 m	442.7	0.1
K	^ 42^K	12.52 h	1524.7	50
La	^ 140^La	40.27 h	1596.2	0.1
Mg	^ 27^Mg	9.5 m	1014.1	50
Mn	^ 56^Mn	2.58 h	1810.7	0.1
Mo	^ 99^Mo	2.75 d	141.0	1
Na	^ 24^Na	15.0 h	1368.4	100
Nd	^ 147^Nd	11.1 d	531.0	1
Ni	^ 58^Co	70.78 d	810.7	1
Rb	^ 86^Rb	18.66 d	1076.7	1
Sb	^ 122^Sb	2.70 d	564.0	0.2
Sc	^ 46^Sc	83.85 d	889.2	0.003
Se	^ 75^Se	120.4 d	264.6	0.1
Sm	^ 153^Sm	1.948 d	103.1	0.04
Ta	^ 182^Ta	115 d	1221.6	0.005
Tb	^ 160^Tb	72.1 d	879.4	0.01
Th	^ 233^Pa	27.4 d	311.8	0.1
Ti	^ 51^Ti	5.8 m	320.0	200
U	^ 239^Np	2.35 d	228.2	0.03
V	^ 52^V	3.76 m	1434.4	1
W	^ 187^W	24.0 h	685.7	0.01
Yb	^ 175^Yb	4.21 d	396.1	0.01
Zn	^ 65^Zn	243.8 d	1115.5	0.5
*IPAA *				
As	^ 75^As	17.9 d	596.0	0.2
Pb	^ 204^Pb	52.1 h	279.0	5
Tl	^ 203^Tl	12.0 d	440.0	0.5
Zr	^ 90^Zr	79.4 h	909.0	0.2

**Table 2:** Results of INAA quality control on IAEA air filter samples (*µ*g g^−1^). The “measured value” is the average of seven determinations on seven different replicates. s.d.: standard deviation; n.d.: not detected.

Element	Measured value	Certified value	Average value	
	Mean ± s.d.	Mean	Mean	2*σ* (%)
As	4.9 ± 0.5	5.6	4.59	43
Au	1.26 ± 0.10	1.15	1.06	21
Ba	43.4 ± 0.5	53.8	39.05	40
Cd	10.6 ± 1.0	9.96	9.8	18
Co	1.3 ± 0.1	1.12	1.18	38
Cr	4.7 ± 0.8	5.6	4.8	13
Cu	51.6 ± 0.5	48.8	44.8	16
Fe	193 ± 17	207.9	200.1	8
Mn	31.2 ± 1.0	31.9	30.1	14
Mo	1.26 ± 0.2	1.14	1.56	70
Se	1.01 ± 0.10	1.06	1.01	11
U	0.78 ± 0.10	1.02	0.99	14
V	8.04 ± 0.35	8.00	7.2	16
Zn	132 ± 18	152	141	12

**Table 3:** Seasonal element concentrations (average, min, and max levels expressed as ng m^−3^) in PM_10_ determined by INAA and IPAA(^a^) in atmospheric particulate sampled in downtown Rome.

Element	Summer			Winter		
	Average	Min	Max	Average	Min	Max
Ag	0.22	0.1	0.5	0.25	0.1	0.6
Al	2900	500	5300	800	200	1600
As^a^	4	1	15	3	1	8
Ba	60	30	120	30	5	70
Br	40	20	70	50	10	140
Ca	2200	800	4200	1200	300	2000
Cd	0.4	0.3	0.9	0.65	0.3	2
Ce	5.6	0.7	10	2	0.2	5
Cl	1300	300	3900	1400	300	4700
Co	0.7	0.4	1.2	0.5	0.1	0.9
Cr	7	4	13	19	2	41
Cs	1.2	0.6	2.2	0.6	0.2	1.3
Eu	0.08	0.04	0.14	0.03	0.01	0.07
Fe	2200	1000	3600	1100	400	2700
Hf	0.36	0.15	0.62	0.13	0.01	0.30
Hg	0.14	0.05	0.36	0.12	0.03	0.20
I	6	3	8	6	3	10
K	1120	50	1900	320	50	1000
La	6	3	11	3	0.3	5
Mg	1090	50	2700	560	200	1200
Mn	40	15	60	35	10	90
Mo	0.5	0.2	0.6	2	0.3	7
Na	1500	300	7000	730	200	3500
Ni	1.7	1	5	6	3	13
Pb^a^	90	40	180	120	40	340
Rb	17	10	30	7	2	15
Sb	2	0.7	4	2	0.7	7
Sc	0.3	0.1	0.5	0.1	0.03	0.2
Se	0.6	0.2	1.1	0.4	0.2	1.3
Ta	0.03	0.01	0.05	0.01	0.005	0.02
Tb	0.055	0.1	0.10	0.024	0.01	0.05
Th	3	1	5	0.7	0.2	2.4
Ti	540	200	1050	265	200	500
Tl^a^	1	0.5	2	1.4	0.5	4
U	0.4	0.1	0.7	0.2	0.06	0.4
V	8	1	15	12	8	23
Zn	70	30	180	110	40	220
Zr^a^	50	10	90	20	5	50

**Table 4:** Grouping of elements in PM_10_ according to the summer/winter ratio seasonal average.

Ratio >2	Ratio ~1	Ratio <1
Al, Ba, Ca, Ce, Cs, Eu, Fe, Hf, K, La, Mg, Na, Rb, Sc, Ta, Tb, Th, Ti, U, Zr	Ag, As, Br, Cl, Co, Hg, I, Mn, Sb, Se, Tl	Cd, Cr, Mo, Ni, Pb, V, Zn

**Table 5:** Synoptic table (mean value, min–max values, and standard deviation) of elements concentration (ng m^−3^) determined in PM_2.5_ in downtown Rome (LOD: limit of detection; *expressed as pg m^−3^; **expressed as *µ*g m^−3^).

Element	PM_2.5_		
	Mean	Min–Max	St. Dev.
As	1.06	0.121–2.76	0.044
Au	0.009	0.000–0.050	0.012
Ba	3.76	1.91–6.45	2.38
Br	17.1	3.20–50.4	13.9
Ce	0.130	0.033–0.335	0.089
Co	0.167	0.077–0.331	0.065
Cr	3.03	1.29–6.40	1.30
Cs	0.047	0.004–0.124	0.037
Eu*	1.14	1.12–1.16	0.029
Fe**	0.074	0.005–0.212	0.059
Hf	0.018	0.006–0.032	0.010
Hg	0.818	0.195–2.12	0.655
La*	22.6	8.73–53.3	10.5
Mo	0.748	0.017–3.04	0.699
Nd	<LOD		
Ni	3.54	1.91–5.82	1.45
Rb	1.82	0.416–3.74	1.07
Sb	3.60	0.690–12.6	3.24
Sc*	3.14	0.208–7.49	2.41
Se	0.567	0.116–1.55	0.415
Sm*	3.88	0.208–7.78	2.13
Th	0.027	0.007–0.041	0.010
W	0.636	0.062–2.86	0.682
Yb	0.011	0.003–0.027	0.007
Zn	58.0	4.78–252	61.3

**Table 6:** Correlation coefficients for the trend of concentrations of analyzed elements present in PM_2.5_.

As	Au	Ba	Br	Ce	Co	Cr	Cs	Fe	Hf	Hg	La	Mo	Rb	Sb	Sc	Se	Sm	Th	W	Zn	
	0.25	−0.80	**0.85**	0.37	**0.85**	0.65	0.50	**0.80**	0.51	−0.35	**0.70**	0.50	0.39	**0.83**	0.40	**0.80**	0.55	0.20	−0.19	0.37	As
		−0.97	0.58	0.51	0.35	0.06	0.54	0.33	**0.73**	0.35	0.01	0.10	**0.77**	0.48	0.01	0.56	0.08	−0.32	−0.19	0.16	Au
			−0.97	−1.00	−0.98	−0.95	−0.89	−0.65	−1.00	−0.43	−0.81	−0.91	−1.00	−0.99	0.62	−0.94	−0.98	−0.98	−0.11	−0.65	Ba
				0.61	**0.87**	0.54	0.57	**0.83**	**0.86**	−0.22	0.57	0.23	0.68	**0.95**	0.35	**0.89**	0.50	0.16	−0.17	0.58	Br
					0.51	0.39	0.29	0.46	0.59	0.15	0.41	0.15	**0.77**	0.64	0.05	0.66	0.10	−0.32	−0.14	0.50	Ce
						0.69	0.53	**0.81**	0.63	−0.22	**0.72**	0.24	**0.78**	**0.90**	0.35	**0.86**	0.53	0.25	0.10	0.60	Co
							0.09	0.67	−0.13	−0.34	0.66	0.43	0.45	0.61	0.29	**0.71**	0.45	0.04	−0.24	0.44	Cr
								0.44	**0.83**	0.15	0.47	0.19	**0.72**	0.60	0.13	0.43	0.19	0.18	−0.20	0.05	Cs
									0.39	−0.38	**0.73**	0.14	0.57	**0.85**	0.65	**0.79**	**0.72**	0.27	−0.29	0.50	Fe
										0.56	0.13	0.48	−0.05	**0.82**	0.26	**0.73**	0.46	0.26	0.01	0.55	Hf
											−0.34	−0.16	0.22	−0.17	−0.41	−0.21	−0.40	−0.37	−0.03	−0.04	Hg
												0.30	0.49	0.64	0.42	0.60	0.55	0.41	−0.15	0.26	La
													0.36	0.27	−0.07	0.40	0.12	−0.31	−0.21	0.03	Mo
														**0.79**	−0.34	**0.76**	−0.29	−0.57	−0.02	0.37	Rb
															0.41	**0.87**	0.53	0.15	−0.13	0.68	Sb
																0.24	**0.89**	0.47	−0.11	0.44	Sc
																	0.44	−0.06	−0.28	0.53	Se
																		0.39	−0.09	0.50	Sm
																			0.24	0.17	Th
																				0.07	W
																					Zn

**Table 7:** Levels (ng m^−3^) of selected elements investigated along four decades in downtown Rome.

	PM_10_		
	1965–78	1989–92	2000–02
As	4.00		1.35
Br	70	50	22
Cd	0.751		0.520
Co	0.498	0.523	0.379
Cr	16	2.3	7.28
Hg	0.131	0.092	1.07
La	2.04	0.803	0.188
Ni	8.01	1.72	4.54
Pb	270	172	92
Sb	1.99	2.13	9.22
Se	0.533	0.091	0.692
Th	0.911	0.723	0.229
V	14	4.82	4.02
Zn	190	28	80

## References

[1] Dockery DW, Speizer FE, Stram DO, Ware JH, Spengler JD, Ferris BG (1989). Effects of inhalable particles on respiratory health of children. *American Review of Respiratory Disease*.

[2] Dockery DW, Pope CA (1994). Acute respiratory effects of particulate air pollution. *Annual Review of Public Health*.

[3] Harrison RM, Yin J (2000). Particulate matter in the atmosphere: which particle properties are important for its effects on health?. *Science of the Total Environment*.

[4] Cao L, Tian W, Ni B, Zhang Y, Wang P (2002). Preliminary study of airborne particulate matter in a Beijing sampling station by instrumental neutron activation analysis. *Atmospheric Environment*.

[5] Avino P, Capannesi G, Rosada A (2008). Heavy metal determination in atmospheric particulate matter by Instrumental Neutron Activation Analysis. *Microchemical Journal*.

[6] Aras NK, Zoller WH, Gordon GE, Lutz GJ (1973). Instrumental photon activation analysis of atmospheric participate material. *Analytical Chemistry*.

[7] Jervis RE, Pringle TG (1988). Aerosol characterization and apportionment using cascade impactors and activation analysis. *Journal of Radioanalytical and Nuclear Chemistry Articles*.

[8] Chutke NL, Ambulkar MN, Aggarwal AL, Garg AN (1994). Instrumental neutron activation analysis of ambient air dust particulates from metropolitan cities in India. *Environmental Pollution*.

[9] Campanella L, Crescentini G, Avino P, Moauro A (1998). Determination of macrominerals and trace elements in the alga Spirulina platensis. *Analusis*.

[10] Avino P, Carconi PL, Lepore L, Moauro A (2000). Nutritional and environmental properties of algal products used in healthy diet by INAA and ICP-AES. *Journal of Radioanalytical and Nuclear Chemistry*.

[11] Orvini E, Speziali M, Salvini A, Herborg C (2000). Rare earth elements determination in environmental matrices by INAA. *Microchemical Journal*.

[12] Rizzio E, Bergamaschi L, Valcuvia MG, Profumo A, Gallorini M (2001). Trace elements determination in lichens and in the airborne particulate matter for the evaluation of the atmospheric pollution in a region of northern Italy. *Environment International*.

[13] Farinha MM, Freitas MC, Almeida SM, Reis MA (2001). Some improvements in air particulate matter analysis by INAA. *Radiation Physics and Chemistry*.

[14] Bergamaschi L, Rizzio E, Valcuvia MG, Verza G, Profumo A, Gallorini M (2002). Determination of trace elements and evaluation of their enrichment factors in Himalayan lichens. *Environmental Pollution*.

[15] Freitas MC, Almeida SM, Reis MA, Oliveira OR (2003). Monitoring trace elements by nuclear techniques in PM10 and PM2.5. *Nuclear Instruments and Methods in Physics Research A*.

[16] Almeida SM, Reis MA, Freitas MC, Pio CA (2003). Quality assurance in elemental analysis of airborne particles. *Nuclear Instruments and Methods in Physics Research B*.

[17] Avino P, Capannesi G, Rosada A (2006). Characterization and distribution of mineral content in fine and coarse airborne particle fractions by neutron activation analysis. *Toxicological and Environmental Chemistry*.

[18] Capannesi G, Diaco L, Rosada A, Avino P (2008). Investigation of trace and ultra-trace elements of nutritional and toxicological significance in Italian potable waters by INAA. *Journal of Radioanalytical and Nuclear Chemistry*.

[19] Capannesi G, Rosada A, Avino P (2009). Elemental characterization of impurities at trace and ultra-trace levels in metallurgical lead samples by INAA. *Microchemical Journal*.

[20] Seccaroni C, Volante N, Rosada A, Ambrosone L, Bufalo G, Avino P (2008). Identification of provenance of obsidian samples analyzing elemental composition by INAA. *Journal of Radioanalytical and Nuclear Chemistry*.

[21] Avino P, Capannesi G, Diaco L, Rosada A (2010). Multivariate analysis applied to trace and ultra-trace elements in Italian potable waters determined by INAA. *Current Analytical Chemistry*.

[22] Capannesi G, Rosada A, Avino P (2010). Radiochemical separation and anti-compton analysis of Ni, Sn, Te and Zn in lead standard reference materials at ultra-trace levels. *Current Analytical Chemistry*.

[23] Buonanno G, Stabile L, Avino P, Vanoli R (2010). Dimensional and chemical characterization of particles at a downwind receptor site of a waste-to-energy plant. *Waste Management*.

[24] Avino P, Capannesi G, Rosada A (2011). Ultra-trace nutritional and toxicological elements in Rome and Florence drinking waters determined by Instrumental Neutron Activation Analysis. *Microchemical Journal*.

[25] Avino P, Santoro E, Sarto F, Violante V, Rosada A (2011). Neutron activation analysis for investigating purity grade of copper, nickel and palladium thin films used in cold fusion experiments. *Journal of Radioanalytical and Nuclear Chemistry*.

[26] Avino P, Capannesi G, Manigrasso M, Sabbioni E, Rosada A (2011). Element assessment in whole blood, serum and urine of three Italian healthy sub-populations by INAA. *Microchemical Journal*.

[27] Capannesi G, Rosada A, Manigrasso M, Avino P (2012). Rare earth elements, thorium and uranium in ores of the North-Latium (Italy). *Journal of Radioanalytical and Nuclear Chemistry*.

[28] Avino P, Capannesi G, Renzi L, Rosada A (2013). Instrumental Neutron Activation Analysis and statistical approach for determining baseline values of essential and toxic elements. *Ecotoxicology and Environmental Safety*.

[29] Vandecasteele C (1991). Activation analysis: present status in relation to other analytical techniques. *Mikrochimica Acta*.

[30] Currie LA (1968). Limits for qualitative detection and quantitative determination: application to radiochemistry. *Analytical Chemistry*.

[31] Djingova R, Kuleff I (2000). Chapter 5 Instrumental techniques for trace analysis. *Trace Metals in the Environment*.

[32] Di Palo L, Focaccia G, Lo Prato E (1967). Il reattore RC-1 ad 1 MW del Centro Studi Nucleari della Casaccia. Caratteristiche generali e programmi di ricerca. *Energia Nucleare*.

[33] Djingova R, Arpadjan S, Kuleff I (1991). INAA and flame AAS of various vegetable reference materials. *Fresenius’ Journal of Analytical Chemistry*.

[34] Locardi E, Sircana S (1967). Distribuzione dell’uranio e del torio nelle vulcaniti quaternarie alcaline del Lazio settentrionale. *Rendiconti Della Società Mineralogica Italiana*.

